# Delineation of *Culicoides* species by morphology and barcode exemplified by three new species of the subgenus *Culicoides* (Diptera: Ceratopogonidae) from Scandinavia

**DOI:** 10.1186/s13071-015-0750-4

**Published:** 2015-03-10

**Authors:** Soren Achim Nielsen, Michael Kristensen

**Affiliations:** Department of Environmental, Social and Spatial Change, Roskilde University, 12.2, 4000 Roskilde, Denmark; Department of Agroecology, Aarhus University, Slagelse, Denmark

**Keywords:** *Culicoides*, COI, DNA barcodes, Morphology, Taxonomy, Bluetongue virus

## Abstract

**Background:**

*Culicoides* biting midges (Diptera: Ceratopogonidae) cause biting nuisance to livestock and humans and are vectors of a range of pathogens of medical and veterinary importance. Despite their economic significance, the delineation and identification of species where only morphology is considered, as well as the evolutionary relationships between species within this genus remains problematic. In recent years molecular barcoding has assisted substantially in the identification of biting midges in the multiple entomological survey projects which were initiated in many European countries following the bluetongue outbreak in 2006–2009. These studies revealed potentially new species and “species-complexes” with large genetic and morphological variability. Here we use molecular barcoding, together with morphological analysis, to study subgenus *Culicoides* Latreille from Scandinavia with focus on three potentially new species.

**Methods:**

Biting midges were collected at various sites in Denmark and Sweden. *Culicoides* specimens were described by variation of a fragment of their cytochrome c oxidase subunit 1 (COI) gene sequence and wing, palp and antennal characters.

**Results:**

It is shown that three new species initially separated by DNA barcoding with mitochondrial COI can be distinguished by morphological characters. In this context a key to Scandinavian subgenus *Culicoides* using wing and maxillary palp characters is presented. The key is including the three new species *Culicoides boyi*, *Culicoides selandicus* and *Culicoides kalix*.

**Conclusion:**

Three new species of *Culicoides* biting midges were identified and could be identified by both molecular and morphological differences. Evaluation of differences between and within taxa of biting midges using COI barcode yielded a rough estimate of species delineation; interspecies differences across *Culicoides* subgenera approaches 20%, whereas intraspecies differences are below 4% and in most cases below 1%.

**Electronic supplementary material:**

The online version of this article (doi:10.1186/s13071-015-0750-4) contains supplementary material, which is available to authorized users.

## Background

The emergence in Europe of bluetongue and Schmallenberg virus, which are both vectored by *Culicoides* (Diptera: Ceratopogonidae) biting midges [1], has increased the interest in these tiny haematophagous insects. Many European countries have implemented entomological surveillance programs as part of contingency plans for these *Culicoides*-borne diseases. *Culicoides* biting midges were thus collected in large parts of Europe from the Swedish Lappland to the Portuguese Azores [2-4]. Many countries have updated their species lists and new species have been discovered of this genus [5]. Additionally, new species emerged when large samples were surveyed; as an example, Switzerland [6-8], Denmark [9,10] and Sweden [2,11].

In the context of a major entomological monitoring program of *Culicoides* in Denmark and Sweden due to the occurrence of bluetongue disease in 2007–08 *Culicoides* specimens were collected by light traps [12]. Most specimens were morphologically identified and some were identified by DNA barcoding, providing an efficient method for species identification of *Culicoides* specimens [9,10,13]. In this context we identified multiple specimens with morphological similarity to *Culicoides pulicaris* (Linnaeus 1758) and *Culicoides punctatus* (Meigen 1804), but with divergent cytochrome c oxidase subunit 1 (COI) barcode sequences [9]. Additionally the amount of published *Culicoides* sequences in GenBank increased steadily as a result of the high activity in the area due to the European bluetongue outbreak and cryptic species seemed to emerge especially within the subgenus *Culicoides* Latreille 1809 [5].

**Figure 1:**
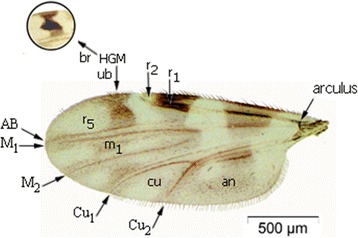
**Wing venation of Culicoides sp.** Wing venation abbreviations: an: anal cell; Cu1, Cu2: first and second cubital veins; M1, M2: first and second median veins; r1, r2: first and second radial cells; r5: radial cell; m1: first medial cell; HGM: Hourglass mark (ub: unbroken-br: broken).

**Figure 2:**
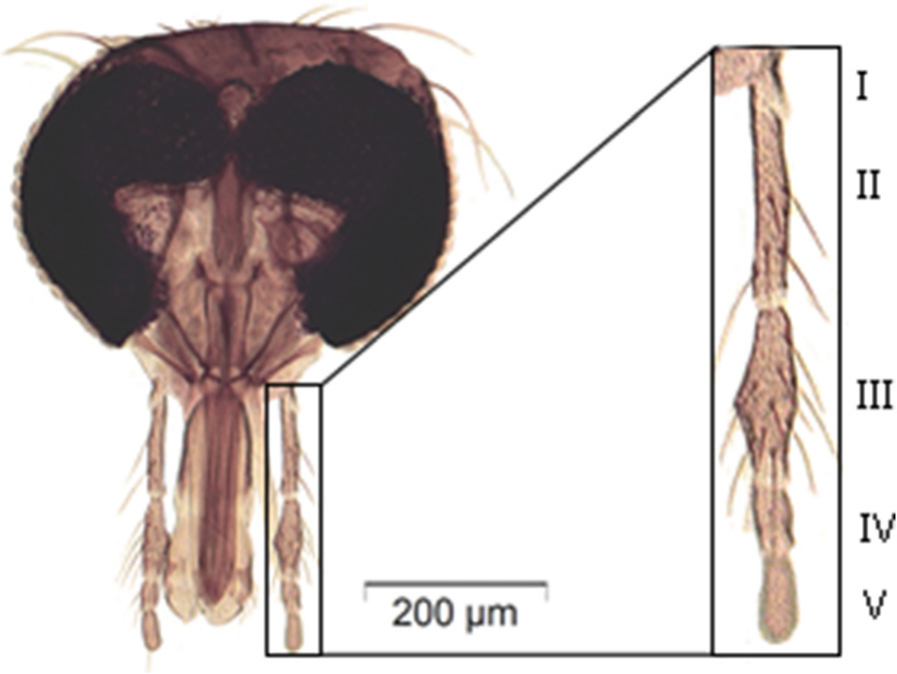
**Head of** ***Culicoides*** **sp. where antennae are removed.** I – V: Maxillary palp segments.

*Culicoides* is a highly diverse genus with more than 1,300 species distributed worldwide [14,15]. One of the subgenera with many species represented in Europe is the subgenus *Culicoides*. The number of species that comprise the subgenus *Culicoides* in the Palearctic region is not known as various authors use the term “species-complexes” with large morphological variability, which combines related taxa and probably overshadows several undescribed species [5,16,17]. Additionally, it is important to demonstrate the geographic distribution of intraspecific morphological and genetic variation of the different species. Following Campbell and Pelham-Clinton [18] the subgenus *Culicoides* is morphologically characterized by wings with dark markings on a light background, and the apical third of the second radical cells are included in a pale area (Figure 1). In the wing cell an hour-glass shaped mark (r_5_) is present. The hour glass shaped cell can be broken or unbroken. The cubital cell (cu) can be with or without a dark spot (Figure 1). In some species that normally lack a spot in the cubital cell, a spot may occur in a minority of specimens of some species e.g. *C. impunctatus* Goetghebuer 1920 and *C. deltus* (Edwards 1939) [*C. deltus* (Edwards) + *C. lupicaris* Downes and Kettle 1952]. The sensorium of the third palp is distributed over many shallow excavations never forming a true pit (Figure 2).

Identification of *Culicoides* to species level is difficult even for specialist taxonomists. It has been shown that the sibling species in species complexes are difficult to distinguish by morphology [16,19]. To overcome this, molecular tools (COI-barcodes or other molecular markers) has been implemented to interpret species of *Culicoides* especially those species implicated in the spread of diseases of domestic animals [11,20-23]. Following barcode identification of specimens to species it may be possible to find new morphological characters or combinations of characters that will assist correct species identification using solely morphological characters. This is desirable when large amounts of *Culicoides* specimens must be identified in the context of ecological studies and will make it possible to identify aberrant specimens which will then be subject to further studies. These specimens are dissected and the head, wings and the terminal abdominal segments are mounted on glass slides for confirmation and documentation of the identified species (voucher) and the rest of the abdomen and thorax are used for molecular identification. This method is semi-destructive where only partial specimens are available for morphological examination. In the future the non-destructive DNA extraction technique described of Bellis et al. [24] can be an alternative that also allows the retention of entire, cleared specimens ready for slide-mounting alongside corresponding DNA data.

In many studies, it is difficult to distinguish between closely-related biting midge species in which the females are apparently identical, which has hampered understanding of exactly which species are involved as vectors. For example in the UK, populations of Obsoletus and Pulicaris group biting midges from different geographical locations was characterized to have different susceptibilities to the same bluetongue virus strain, which may reflect varying susceptibilities between different species (*sensu stricto*) [25]. It is therefore necessary with the help of morphological and molecular biological methods to be able to distinguish the different species.

In Denmark and Sweden the subgenus *Culicoides* has until now been represented by six species; *C. pulicaris*, *C. punctatus*, *C. impunctatus*, *C. deltus*, *C. grisescens* Edwards 1939, and *C. newsteadi* Austen 1921(syn. *C. halophilus* Kieffer 1924) ([26] *Culicoides fagineus* Edwards 1939 has been removed from the checklist by revision). Barcodes from multiple specimens from Scandinavian subgenus *Culicoides* species differentiated into eight unique clusters, including the five common Palaearctic species *C. punctatus*, *C. pulicaris*, *C. impunctatus*, *C. grisescens* and *C. deltus*. Additionally, this study confirmed the existence of a Scandinavian *C. newsteadi* (which was proposed to be *C. halophilus*) and presented three additional distinct barcode groups, which were proposed to be new taxa [9]. This study describes and quantifies morphologically differences of the six common species of the subgenus *Culicoides* known from Scandinavia including the three new species. The differences are visualised in tables with antenna, maxillary palp, wing and spermatheca characters and including an identification key (based on a combination of wing and maxillary palp characters).

## Methods

### Sampling and identification of biting midges

**Figure 3:**
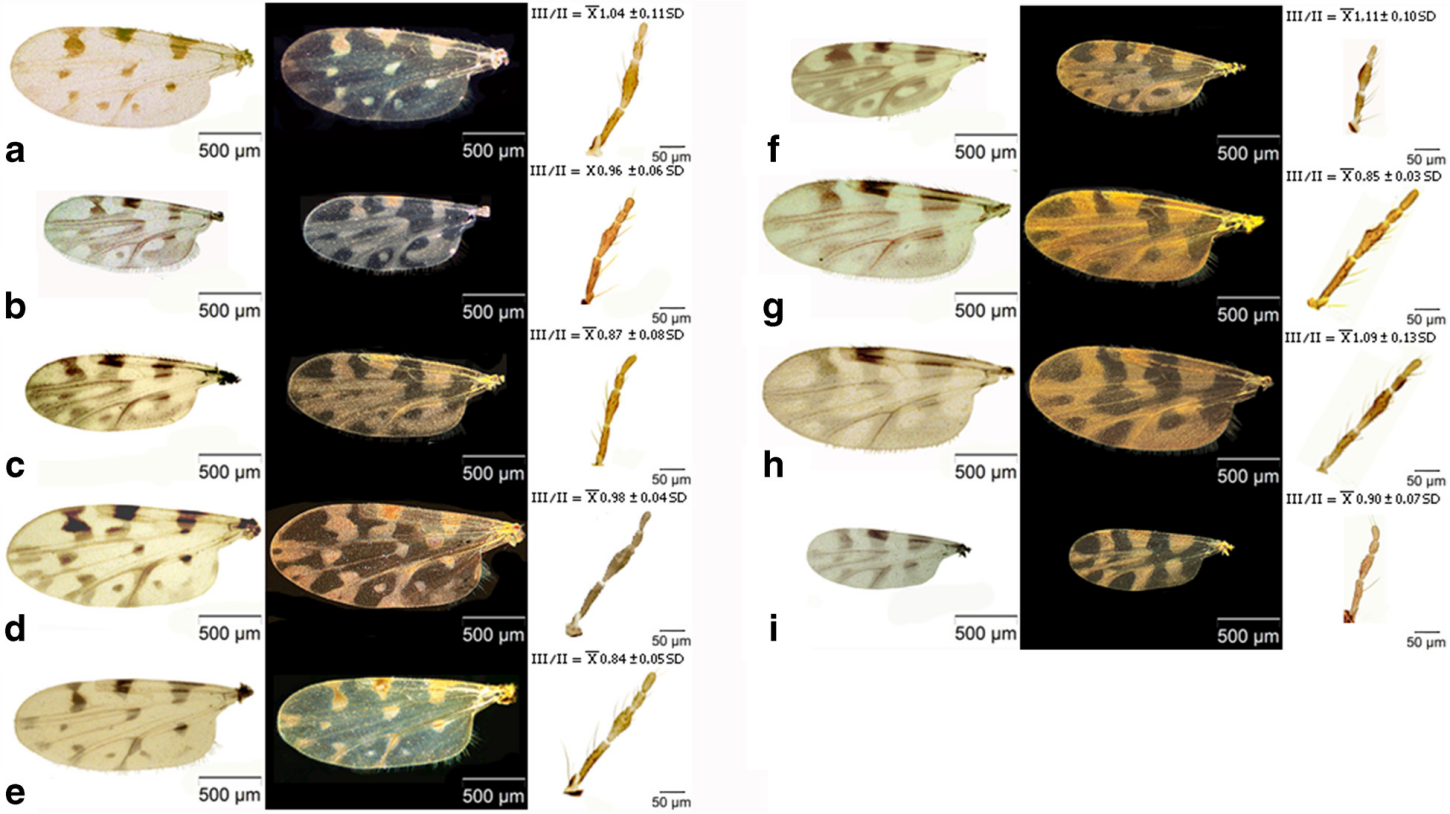
**Wings (bright and dark field images) and maxillary palpae of female** ***Culicoides*** **(** ***Culicoides*** **)** **from Scandinavia. a:** *C. boyi*; **b:** *C. selandicus*; **c:** *C. kalix*; **d:** *C. punctatus*; **e:** *C. pulicaris*; **f:** *C. newsteadi*; **g:** *C. deltus*; **h:** *C. grisescens*; **i:** *C. impunctatus*.

Biting midges were collected from July to October at various sites in 2008 in Denmark [9] and in 2007 in Sweden [2] (Table 1). The samplings were performed for one night and the insects were collected in water added to a few drops of detergent. The insect material was removed by filtration and transferred to 70% ethanol. *Culicoides* samples were morphologically identified under a stereomicroscope according to the wing and palp characters (Figure 3a - i) consulting different keys [17,18,27-31].

**Table 1:** **Biting midges were collected from July to October at various sites in 2008 in Denmark (DK) and in 2007 in Sweden (SE)**

**Culicoides Species**	**Locality**	**Coordinate**
***C. boyi***	Aalestrup, DK	56°40′5.13″N, 09°28′53.92″E
**-**	Nibe, DK	56°53′0.25″N, 09°50′49.79″E
***C. selandicus***	Næstved, DK	55°10′47.49*″N*, 11°50′14.77″E
***C. punctatus***	Aalestrup, DK	56°40′5.13″N, 09°28′53.92″E
***C. pulicaris***	Aalestrup, DK	56°39′8.19″N, 09°34′8,89″E
***C. newsteadi***	Ølstykke, DK	55°48′30.27″N,12°09′12,99″E
***-***	Fuglebjerg, DK	55°17′38.12″N, 11°32′23.27″E
***-***	Næstved, DK	55°11′20.15″N, 11°47′58.61″E
***-***	Nibe, DK	56°54′20.16″N, 09°37′24.06″E
***C. deltus***	Randbøl, DK	55°41′56.27″N, 09°15′8.01 ″E
***C. grisescens***	Tarm, DK	55°51′7.24″N, 08°46′1.69″E
***C. impunctatus***	Randbøl, DK	56°40′5.13″N, 09°28′53.92″E
***C. kalix***	Kalix, SE	65°44′ 45.13″N, 23° 03′55.62″E

For documentation of the identification, the head, wings and the posterior abdominal segments were removed from the female individuals and slide mounts were made (Unfortunately, the posterior part of the abdomen of *C. selandicus* was not removed from the females and stored on slide mounts before the rest of the animal was transferred to DNA analysis. Therefore, there is no data for spermatheca for this species). The remaining parts were transferred to a microcentrifuge tube with 96% ethanol for later DNA analysis.

Under a stereomicroscope at 12-15× magnification, the head, wing and tip of abdomen were placed in a drop of Euparal (Carl RothGmbH + Co, Karlsruhe, Germany) on a slide after which both antennae were removed from the head with microneedles and finally covered with a cover slip. Subsequently the lengths of every palp and flagellar segments, wings (from arculus to tip) and spermatheca were observed under an Olympus CX41microscope (Olympus) equipped with an Olympus SC30 digital camera. Measurements of the different parts of the specimens were performed using the CellD analyzing software. Following Campbell and Pelham-Clinton [18] antennae ratio (AR: 11–15 antennal segments divided with segments 3–10) and palp ratio (PR: length of segment 3 divided with greatest breath) were calculated.

The significance of differences between measurements was determined by multiple comparison test after Kruskal–Wallis (P_K-W_ <0.05) followed by a Conover-Inman test for all pairwise comparisons.

In accordance with section 8.5 of the ICZN’s International Code of Zoological Nomenclature, details of the new species have been submitted to ZooBank with the life science identifier (LSID) zoobank.org:pub:AFF7422D-EC51-4B8A-AA32-BD4F5B8F8C78

### Comparison of *Culicoides* COI barcodes

The specimens used for morphological measurements were all COI barcoded, uploaded to NCBI GenBank and published earlier in the context of a project evaluating host preference of biting midges [9,10,13].

The COI barcode (a fragment of the mitochondrial cytochrome C oxidase subunit 1) of individual biting midges was collected from NCBI GenBank using CLC Main Workbench (CLCbio, Aarhus, Denmark). Their GenBank accession numbers can be found in Additional file 1: Table S1 with reference to their geographic origin.

Interspecies differences of *Culicoides* COI sequences published in GenBank were calculated by pairwise comparisons (CLCbio). There is variation among individual submissions and the interspecies divergence is thus a range of values, which are presented in Table 2. Three numbers calculated are: a) SNP-Sites: Number of variable sites in the 472 bp COI barcode region, b) Var: The highest intraspecific difference observed in pairwise comparisons, and c) Div: Highest divergence from type-species *C. punctatus* (GenBank AM236733) observed in pairwise comparisons.

**Table 2:** **Comparison of** ***Culicoides*** **COI barcode sequences of three Palaearctic subgenera;** ***Avaritia*** **,** ***Culicoides*** **and** ***Monoculicoides***

**Species**		**SNP-sites**	**Var**	**Div**	**Comments**
	**N**	**N**	**%**	**%**	
**Subgenus:** ***Avaritia***					
***C. chiopterus***	32	31	2.3	18	
***C. dewulfi***	53	11	0.85	21	
***C. imicola***	80	45	2.8	18	
***C. obsoletus***	127	42	3.8	17	
***C. scoticus***	89	24	1.7	17	
**Subgenus:** ***Culicoides***					
***C. deltus***	12	5	0.85	17	
***C. fagineus*** **F1**	3	2	0.42	19	*C. fagineus* F1 and F2 diverge 8.5% from each other.
***C. fagineus*** **F2**	4	7	1.5	19	
***C. flavipulicaris***	2	1	0.21	17	
***C. grisescens***	22	10	1.3	17	*C. grisescens* G1 and G2 diverge 11% from each other.
***C. grisescens*** **G2**	6	12	1.9	17	
***C. impunctatus***	20	7	0.64	18	
***C. lupicaris***	15	9	0.42	16	
***C. lupicaris*** **L2**	8	3	0.42	15	*C. lupicaris* L2 diverge 14% from *C. lupicaris.*
***C. newsteadi***	9	1	0.21	16	
***C. newsteadi*** **N1**	1	n.a.	n.a.	16	*C. newsteadi* N1 diverge 14-17% from *C. newsteadi*, N2, N3, N4 and N5.
***C. newsteadi*** **N2**	5	8	1.5	17	*C. newsteadi* N2 diverge 14-16% from N3, N4 and N5.
***C. newsteadi N3*** **(** ***C. halophilus*** **)**	17	5	0.64	19	*C. newsteadi* N3 diverge 14-16% from *C. newsteadi*, N4 and N5.
***C. newsteadi*** **N4 (** ***kalix*** **)**	10	0	0.00	13	*C. newsteadi* N4 diverge 14% and 5.9% from N4 and N5, respectively.
***C. newsteadi*** **N5 (dk3)**	5	6	1.3	15	*C. newsteadi* N5 and *C. newsteadi* diverge 15% from each other*.*
***C. pulicaris***	57	16	0.63	17	*C. pulicaris* P3 diverge 10% from *C. pulicaris.*
***C. pulicaris*** **P3**	2	1	0.21	15	
***Culicoides*** **dk1**	9	0	0.00	18	*Culicoides* dk1 diverge 17-18% from *C. pulicaris* and P3, respectively.
**CH**	12	23	4.6	15	*Culicoides* CH diverge 8-9% from *C. pulicaris* and P3
***C. punctatus***	61	32	0.63	0	
***C. subfagineus***	3	2	0.42	17	
**Subgenus:** ***Monoculicoides***					
***C. nubeculosus***	12	3	0.64	19	
***C. puncticollis***	20	0	0.00	19	
***C. riethi***	16	1	0.21	19	
***C. stigma***	6	0	0.00	17	

Species are grouped according to their subgenus status; species names are those used in the papers where the sequences are published. N: Number of *Culicoides* sequences which are >95% homologous. SNP-Sites: Number of variable sites in the 472 bp COI barcode region. Var: The highest intraspecific difference observed in pairwise comparisons. Div: Highest divergence from type-species *C. punctatus* (GenBank AM236733) observed in pairwise comparisons. n.a.: not applicable.

## Results and discussion

### Species delineation of *Culicoides*

This is not a comprehensive elucidation of global species delineation of the 1,300 genus *Culicoides* species, but an attempt to show one way of creating order in the complex situation that is surrounding the genus *Culicoides*. An initial simple way of evaluating the delineation of *Culicoides* species by DNA barcoding is using pairwise comparisons of sequences of European specimens. Within the subgenus *Avaritia* Fox 1955 the difference within species varies from 3.8% (N = 127) in *C. obsoletus* (Meigen 1818) to 0.85% (N = 53) in C. *dewulfi* Goetghebuer 1936 (Table 2). The low intraspecies variation in *Avaritia* is remarkable, particularly in light of the large geographical spread of *C. scoticus* Downes and Kettle 1952, which show only 1.7% (N = 89) variation even though sequences originate from specimens collected from Spain to Northern Sweden. The geographic origin of all sequences used for comparison can be found in Additional file 1: Table S1. Interspecies comparisons of the four *Avaritia* species with the *Culicoides* type-species *C. punctatus* showed divergences from 17% to 21% (Table 2).

Likewise, comparing the sequence from subgenus *Monoculicoides* Khalaf 1954, very little intraspecies variation is observed from 0% (N = 20) in *C. puncticollis* (Becker 1903) to 0.64% in *C. nubeculosus* (Meigen 1830) (N = 10) (Table 2). This very low variation could be explained by specimens all originating from Scandinavia as well as low sample size. Interspecies comparisons of the four *Monoculicoides* species with *C. punctatus* showed divergences from 17% to 19% (Table 2).

An overview of European interspecies variation can be found in Additional file 2: Table S2, where all available species are compared to *C. punctatus*. Interspecies variation is between 16% and 21%.

Intra- and interspecies comparisons of all the sequences assign to the subgenus *Culicoides* showed a similar pattern. Intraspecies variation within *C. pulicaris* (N = 57) and *C. punctatus* (N = 61) was in both cases 0.63% cases with sequences from specimens collected from a wide geographic area. The interspecies difference of *C. pulicaris* and *C. punctatus* was 17% (Table 2). An interspecies comparison of the 21 cryptic or putative subgenus *Culicoides* species listed in Table 2 showed variation from 13% to 19%. This is lower than the above mentioned variation from the European *Culicoides* species, and is probably due to the fact that the sequence use for comparison is from the type-species *C. punctatus*, i.e. it is from within the same subgenus.

Earlier studies showed that the molecular COI barcoding method successfully supported the identification of morphologically pre-identified *C. punctatus*, *C. pulicaris*, *C. deltus*, *C. grisescens*, *C. newsteadi* and *C. impunctatus* specimens to species [9]. Additionally, three groups of specimens with unknown COI DNA barcodes were identified [9]. Our study gave molecular evidence for retaining *C. halophilus* (identical to *C. newsteadi N3* described by Pages et al. [5]) as a true species as well as suggesting the presence of three new species based on the unknown barcodes mentioned above [9] including *C. newsteadi* N4 described by Pages et al. [5] and recorded as divergent in a neighbor-joining phylogenetic tree [11].

The observed interspecific divergence values correspond well with other barcoding studies, e.g. a study of >1,300 Lepidoptera species from North-America showed a mean divergence of 7.7% between species. The intraspecific divergence averaged 0.43% even though comparisons involved populations 500–2,800 km apart. Although most species possessed low intraspecific divergence some taxa included barcode groups with more than 2% sequence divergence [32]. This probably reflects overlooked species pairs as was shown for a tentative species with 3.8% intraspecific divergence, which subsequently revealed differences in morphology and ecology [33,34]. It should also be noted that in a few cases barcodes were shared between apparently distinct Lepidoptera species [32]. In study of Irish solitary bees involving 55 species the intra- and interspecies differences were more similar. Pairwise comparisons of COI sequences showed a distinct break between interspecies and intraspecies genetic distance around 1% although variation at this point was continuous [35]. The interpretation of barcode data is not yet at a mature state and collection and comparison of data from many different genera and taxa should still be in focus. Additionally, species delineation by analyz single locus data can be used for primary species identification, but not for “in depth” phylogenetic analysis [36].

In conclusion, interspecies differences across *Culicoides* subgenera using COI barcode approaches 20%, whereas intraspecies differences are below 4% and in most cases below 1%.

### Three new *Culicoides* species

Lassen et al. [9] presented three groups of specimens: *Culicoides* dk1 with a COI barcode diverging by 14% to 17% from other subgenus *Culicoides* species and *Culicoides* Kalix and *Culicoides* dk3, which diverged by 5.9% from each other and showed 13% to 18% divergence in COI barcode to subgenus *Culicoides* specimens (Table 2). Based on a phylogenetic tree clearly separating the three species from other *Culicoides* species [9] as well as the above described species delineation of *Culicoides* species, we claim the existence of three new species. *Culicoides* dk1 is named *Culicoides boyi* Nielsen & Kristensen as a tribute to Boy Overgaard Nielsen an outstanding Danish entomologist from Aarhus University. *Culicoides* dk3 is named *Culicoides selandicus* Nielsen & Kristensen and *Culicoides* Kalix is named *Culicoides kalix* Nielsen & Kristensen after their geographic origin in Denmark and Sweden, respectively.

### Recognition and separation of the three new species belonging to the subgenus *Culicoides* using a stereomicroscope

*Culicoides boyi* can easily be confused with *C. pulicaris*, but can be separated from it by having more well-defined and less extensive dark wing-marks. This is especially visible using a stereomicroscope. Furthermore, the hour-glass mark in r_5_ is broken like by *C. selandicus*, *C. kalix* and *C. punctatus,* but can be separated from those by a lack of eye-spots at the tip of M_1_ and M_2_ (*C. punctatus* and *C. selandicus*), as well as less widespread dark areas on the wing as compared to *C. selandicus* and *C. kalix* (Figure 3). Likewise, *C. boyi* differs from all the species belonging to subgenus *Culicoides* at the long third antennal segment. The third segment ratio is significantly different from all other species (Table 3).

**Table 3:** **Measurement (average ± standard deviation) of the length and female antennae (μm)**

**Species**	**N**_**1**_	**N**_**2**_	**Total length of flagellum (μm)**	**Antennal ratio (AR)**	**Ratio third segment**
***C. pulicaris***	10	18	742 ± 26^C^	1.09 ± 0.03^B^	1.52 ± 0.07^AB^
***C. punctatus***	10	19	684 ± 43^F^	1.14 ± 0.05^AD^	1.51 ± 0.08 ^AB^
***C. boyi***	11	10	746 ± 44^C^	1.03 ± 0.04^C^	1.78 ± 0.07 ^C^
***C. deltus***	5	10	794 ± 45^A^	1.11 ± 0.02^A^	1.50 ± 0.10^AB^
***C. newsteadi***	5	9	591 ± 45^E^	1.04 ± 0.05^C^	1.53 ± 0.15 ^AB^
***C. selandicus***	4	7	616 ± 10^E^	1.12 ± 0.04^AD^	1.56 ± 0.11^B^
***C. impunctatus***	6	11	552 ± 33^D^	1.03 ± 0.05^C^	1.61 ± 0.16^B^
***C. grisescens***	5	10	849 ± 41^A^	1.21 ± 0.09^D^	1.57 ± 0.14^B^
***C. kalix***	5	10	646 ± 14^B^	1.13 ± 0.04^AD^	1.46 ± 0.06^A^

Calculation of the antenna ratio (AR: complete length of the apical five segments of the flagellum (11–15) divided by the length of the eight basal segments (3-10)) and calculation of the third segment ration (length/width). N_1_ = number of specimens; N_2_ = number counted. Means with the same letters are not significant different.

*Culicoides selandicus* and *C. kalix* can be confused with *C. newsteadi* due to the extensive dark wing-markings and broken hour-glass mark. However, both species can be distinguished from *C. newsteadi* since they have only one dark mark in m_1_ where *C. newsteadi* has two. Additionally, both species have a more slender and longer third palp segment compared to *C. newsteadi* (Figure 3, Table 4).

**Table 4:** **Measurement (average ± standard deviation) of the length of female maxillary palp (μm) segments 2 to 5 and calculation of the palp ratio (P-R)**

**Species**	**N**_**1**_	**N**_**2**_	**2**	**3**	**4**	**5**	**Total (μm)**	**P/R**	**P 3/2**
***C. pulicaris***	10	19	99	83	36	38	255.6 ± 14.3^D^	2.9 ± 0.2^A^	0.84 ± 0.05^C^
***C. punctatus***	10	20	83	82	34	35	234.3 ± 15.9^F^	2.9 ± 0.2^A^	0.98 ± 0.04^B^
***C. boyi***	11	21	86	89	36	35	245.1 ± 17.7 ^E^	2.9 ± 0.3^A^	1.04 ± 0.11^E^
***C. deltus***	5	10	109	93	40	45	288.1 ± 22.4^B^	3.0 ± 0.1^A^	0.85 ± 0.03^C^
***C. newsteadi***	5	9	66	74	25	29	193.6 ± 18.7 ^C^	2.6 ± 0.2^D^	1.11 ± 0.10^D^
***C. selandicus***	4	8	80	77	30	30	216.8 ± 8.6^A^	3.2 ± 0.3^B^	0.96 ± 0.06^B^
***C. impunctatus***	6	12	64	58	30	33	184.5 ± 16.0^C^	2.9 ± 0.2^A^	0.90 ± 0.07^A^
***C. grisescens***	5	10	104	113	44	45	305.7 ± 16.3^B^	3.9 ± 0.3^BC^	1.09 ± 0.13^ED^
***C. kalix***	5	10	75	65	31	41	212.4 ± 4.0^A^	2.9 ± 0.2^A^	0.87 ± 0.08^CA^

PR: Length of segment 3 divide by width. P 3/2: Length of third segment divided by length of second segment of the maxillary palp. N_1_ = number of specimens; N_2_ = number counted. Means with the same letters are not significant different.

*Culicoides selandicus* can be distinguished from *C. kalix* by a higher P 3/2 ratio (Figure 3, Table 4). *Culicoides selandicus* is a smaller species compared to *C. kalix* and the outline of wings is more rounded (Figure 3, Table 5).

**Table 5:** **Measurement (average ± standard deviation) of the length of female wings and spermatheca**

**Species**		**Length of wing (μm)**		**Lengths of spermatheca (μm)**			**Spermatheca ratio (S/R)**		**Head proboscis ratio (H/P)**
	**N**_**2**_		**N**_**1**_	**1**	**2**	**3**		**N**_**1**_	
***C. pulicaris***	19	1626 ± 67^C^	10	74 ± 5	61 ± 5	22 ± 4	1.21 ± 0.08^A^	10	1.19 ± 0.04 ^B^
***C. punctatus***	20	1519 ± 10^B^	9	74 ± 7	62 ± 4	19 ± 3	1.19 ± 0.08^A^	10	1.19 ± 0.07 ^B^
***C. boyi***	22	1641 ± 10^C^	5	74 ± 6	68 ± 6	23 ± 4	1.05 ± 0. 3^B^	11	1.29 ± 0.07 ^A^
***C. deltus***	10	1788 ± 12 ^A^	5	72 ± 4	67 ± 5	28 ± 5	1.07 ± 0.05^B^	5	1.19 ± 0.05 ^B^
***C. newsteadi***	10	1291 ± 12^D^	4	69 ± 6	66 ± 4	19 ± 6	1.04 ± 0.02^B^	5	1.32 ± 0.13 ^A^
***C. selandicus***	7	1339 ± 33 ^D^	ND	ND	ND	ND	ND	4	1.16 ± 0.06 ^CB^
***C. impunctatus***	9	1239 ± 13 ^D^	5	56 ± 7	52 ± 10	15 ± 6	1.10 ± 0.11^B^	6	1.30 ± 0.04 ^A^
***C. grisescens***	10	1841 ± 12 ^A^	5	87 ± 3	83 ± 1	33 ± 3	1.05 ± 0.02^B^	5	1.03 ± 0.04 ^C^
***C. kalix***	10	1423 ± 39 ^E^	5	75 ± 8	64 ± 6	20 ± 4	1.17 ± 0.08^A^	5	1.29 ± 0.07 ^A^

Spermatheca ratio (S/R). The ratio of head divide by proboscis (H/P). N_1_ = number of specimens; N_2_ = number counted. ND = not determined. Means with the same letters are not significant different.

### Morphological identification of subgenus *Culicoides* females

To identify the subgenus *Culicoides* females under a stereomicroscope it is necessary to combine several characters. The results are best presented as an identification key. The discriminating characters are wing markings and length and shape of segments of the maxillary palp.

Key to females of the subgenus *Culicoides* in Scandinavia:

**(1a).** Wings with a spot in cell cu …….……………….…………………………………………………………………….**(2)**

**(1b).** Wings without a spot in cell cu……………….....**(9)**

**(2a).** Third segment of the maxillary palp longer than the second segment, or third segment and second segment of the same length. Wings with dark hour-glass mark in cell r_5_ broken above the longitudinal fold above M_1_. …..…………………………………………………………….**(3)**

**(2b).** Third segment of the maxillary palp shorter than the second segment. Wings are with a dark hour-glass mark in cell r_5_ broken or unbroken above the longitudinal fold above M_1_. .…………………………………………………**(6)**

**(3a).** Dark areas on the wing are extensive. Dark areas in wings surrounding vein M_1_ (Figure 3b, f)……………………………………………………………………..**(4)**

**(3b).** Dark areas on the wings are less extensive. No dark areas in wings surrounding vein M_1_ (Figure 3a, d)…………………………………………………………………….**(5)**

**(4a).** Wings are with two dark marks in cell M_1_. The hour-glass mark in r_5_ is broad and roughly square in outline. Third segment of the maxillary palp is longer than the second segment. …………………………***newsteadi*Austen**

**(4b).** Small species (Table 5). Wings are with only one dark mark in cell M_1_. Third and second segment of the maxillary palp are of same length. Dark areas in wings surrounding vein M_1_, sometimes with small pale spots at the tips of veins M_1_ and M_2_.………………………………………………………….***selandicus*Nielsen & Kristensen**

**(5a)**. Wings are with small pale spots at tips of veins M_1_ and M_2_ and Cu_1_. The hour-glass dark mark in the middle of cell r_5_ is broken and broadest above the longitudinal fold above M_1_. Third and second segment of the maxillary palp are of the same length. …………………………………………......***punctatus*(Meigen)**

**(5b).** No pale spots at the tips of vein M_1_, M_2_ and M_3_. Wings are with a dark hour-glass mark in the middle of cell r_5_ broadest above the longitudinal fold above M_1_. Most of the specimens have a spot in cell cu but about one-third has only a very small or no spot in this cell ….…………………………***boyi*Nielsen & Kristensen (part)**

**(6a).** Wings are with the dark hour-glass mark in cell r_5_ broken and broadest above the longitudinal fold above M_1_ or hour-glass mark is unbroken with continuous outline and equal widths above and at the longitudinal fold above vein M_1_ (Figure 2e)……………………………………**(7)**

**(6b).** Wings are with the dark hour-glass mark in cell r_5_ unbroken and broadest at the longitudinal fold above vein M_1_ …………………………….…………………….……..**(8)**

**(7a).** Small species (Table 5). The wings with dark hour-glass mark in r_5_ broken and broadest above the longitudinal fold above vein M_1_. The dark areas in wings are extensive and surrounding vein M_1_ and M_2._ ……………….…………………***kalix*Nielsen & Kristensen**

**(7b).** Large species Table 5). Wings with the dark hour-glass mark in cell r_5_ with continuous outline and equal widths above the longitudinal fold and at the fold above vein M_1_ …………………………………***pulicaris*(L.)**

**(8a).** Small species (Table 5). Wing markings are vague but sharply defined. The hour-glass mark in cell r_5_ is unbroken and broadest at vein M_1_. The hour-glass mark in cell r_5_ is skewed by more than two thirds in the lower portion (Figure 3). A small spot in cu. The shape of the third segment of the maxillary palp is rhomboid ………...............................***impunctatus*Goetghebuer (part)**

**(8b).** Large species (Table 5). Wings are with extensive vaguely defined dark markings usually with a spot in cell cu. The hour-glass mark is unbroken. Third segment of the maxillary palp is shorter than the second segment. Sensorium usually dispersed over numerous larger excavated areas ………………………………………………… ***deltus*Downes and Kettle (part)**

**(9a).** Small species (Table 5). The hour-glass mark in cell r_5_ is unbroken and skewed by more than two thirds in the lower portion (Figure 3). The shape of the third segment of the maxillary palp is rhomboid .…….………..…...................................... ***impunctatus*Goetghebuer (part)**

**(9b).** Large species (Table 5). The shape of hour-glass mark broken or unbroken and the shape of the third palp segment not rhomboid …………………………….. **(10)**

**(10a).** Wings are with dark distinct markings on a light and fainter wing surface. The dark hour-glass mark in the middle of cell r_5_ is broken and broadest above the longitudinal fold above the longitudinal fold above cell M_1_ .......………………… ***boyi*Nielsen & Kristensen (part)**

**(10b).** Wings are with dark vaguely defined markings (Figure 3g, h). The dark hour-glass mark in cell r_5_ unbroken and of broadest at the longitudinal fold above vein M_1_ ………………………………………………………. **(11)**

**(11a).** Wings are with extensive, but vaguely defined, dark markings. The dark stain from the edge of the wing in the anal cell follows that edge even in the distal part. Third segment of the maxillary palp is shorter than the second segment. Sensorium usually dispersed over more numerous and smaller excavated areas in the enlarged middle part of the third segment of the maxillary palp. Wings without spot in cell cu………….…………………………………………………………… ***deltus*Edwards (part)**

**(11b).** Wings markings are very vague. Third segment of the maxillary palp is of same length or longer than the second segment. Third segment of the maxillary palp is very long and narrow ………………………………………..…………………………………………… ***grisescens*Edwards**

### Description of *Culicoides boyi sp. nov*

Up till now only females of this species are known and therefore the following characteristics only apply to females.

The length of the wing is 1,641 ± 10 μm (Table 5). The shape of the dark hour-glass formed mark in the middle of cell r_5_ is broken and is broadest above the longitudinal fold above M_1_, in contrast to *C. pulicaris* where the hour-glass formed mark is with continuous outline and equal widths above the longitudinal fold and at the fold above vein M_1_ (Figure 3a). Furthermore *C. boyi* can be distinguished from *C. pulicaris* by more defined and less extensive dark wing spots. Seen in the stereo microscope the dark wing markings stands distinctly on a light, more faint wing surface and are more light brownish than in *C. pulicaris*. In most of the specimens a spot is present in the cubital cell, but about one-third of the specimens have only a very small or no spot.

The eyes are contiguous and the length of contact divided with the diameter of one ocellus (FV/O ratio) is 1.5 ± 0.2 (Table 6). The length of antennal flagellum, 746 ± 44, is of the same length as in *C. pulicaris* (Table 3). Antennal ratio (AR) (1.03 ± 0.04) is significantly lower than in *C. pulicaris* (Table 3). In *C. boyi* the third antennal segment is long and slender, and the 3A-ratio (length divided by width) is 1.78 ± 0.07, which is significantly different compared to the other species of this subgenus (Table 3). The number of sensillae is of the same magnitude as in *C. pulicaris* (Table 7). The length, form and palp ratio (PR) only differ from *C. newsteadi*, *C. selandicus* and *C. grisescens* (Table 4), whereas the ratio P3/P2 (third segment of the maxillary palp divided by second segment) is significantly different from all other species except *C. grisescens* (P_K-W_ <0.05) (Table 4). There are no significant differences between *C. boyi* and *C. pulicaris* in the ratio of mandibular and maxilla teeth (M/M) (Table 6). The head/proboscis ratios show that *C. boyi* is significantly different from *C. pulicaris*, *C. punctatus*, *C. deltus*, *C. selandicus* and *C. grisescens* (P_K-W_ < 0.05) (Table 5).

**Table 6:** **The number of mandibular and maxillary teeth (average ± standard deviation) and the ratio M/M of mandibular vs. maxillary teeth**

**Species**	**N**_**1**_	**N**_**2**_	**Mandibular teeth**	**N2**	**Maxillary teeth**	**Ratio M/M**	**Fronto-vertex/ocellus**
***C. pulicaris***	10	18	16.7 ± 1.2^C^	17	19.5 ± 1.1^C^	1.17 ± 0.08^A^	1.2 ± 0.3 ^AC^
***C. punctatus***	10	20	15.7 ± 1.2 ^B^	18	19.6 ± 1.0^C^	1.26 ± 0.14 ^B^	0.9 ± 0.5 ^C^
***C. boyi***	11	21	15.1 ± 1.2 ^B^	19	17.1 ± 1.4 ^E^	1.13 ± 0.10 ^A^	1.5 ± 0.2 ^BDF^
***C. deltus***	5	10	15.8 ± 0.6 ^CB^	7	18.1 ± 1.5 ^B^	1.15 ± 0.08 ^A^	1.7 ± 0.4^BD^
***C. newsteadi***	5	10	13.4 ± 1.4 ^A^	8	15.9 ± 1.5 ^A^	1.18 ± 0.20 ^AC^	0.3 ± 0.3 ^E^
***C. selandicus***	4	7	15.0 ± 1.0 ^B^	8	19.6 ± 1.5 ^C^	1.31 ± 0.12 ^BC^	1.5 ± 0.8^AD^
***C. impunctatus***	6	11	13.7 ± 1.0 ^A^	9	15.0 ± 0.9 ^A^	1.12 ± 0.10 ^A^	1.9 ± 0.3^B^
***C. grisescens***	5	10	15.2 ± 1.2 ^B^	10	22.0 ± 0.7 ^D^	1.46 ± 0.13^C^	0 ± 0.0^E^
***C. kalix***	5	10	12.80 ± 0.6 ^A^	10	14.9 ± 1.5^A^	1.17 ± 0.12 ^A^	1.2 ± 0.3^ACF^

The ratio of fronto-vertex divided by ocellus. N_1_ = number of specimens; N_2_ = number counted. Means with the same letters are not significant different.

**Table 7:** **Distribution of antennal sensilla on segments 3–15**

**Species**	**N**_**1**_	**N**_**2**_	**3**	**4**	**5**	**6**	**7**	**8**	**9**	**10**	**11**	**12**	**13**	**14**	**15**	**Total**	**Min-Max**
***C. pulicaris***	10	17	3.29	0.00	0.00	0.00	0.00	0.00	0.00	0.00	1.35	1.12	1.71	3.59	4.41	15.47 ± 1.94^A^	12-19
***C. punctatus***	10	19	3.37	0.00	0.05	0.00	0.00	0.00	0.16	0.00	1.00	1.00	1.11	2.00	3.32	12.00 ± 0.94^C^	10-14
***C. boyi***	11	17	4.94	0.00	0.00	0.00	0.00	0.00	0.00	0.00	1.53	1.47	1.88	2.29	2.59	14.71 ± 1.26^A^	12-16
***C. deltus***	5	10	3.40	0.00	0.00	0.00	0.00	0.00	0.00	0.00	1.90	1.90	2.00	3.70	4.00	17.10 ± 2.47^E^	12-20
***C. newsteadi***	5	9	2.89	0.00	0.00	0.00	0.00	0.00	0.00	0.00	0.33	0.89	0.89	0.78	1.89	7.70 ± 0.67^D^	6-8
***C. selandicus***	4	7	4.10	0.00	0.00	0.00	0.00	0.00	0.00	0.00	1.00	1.00	1.00	2.29	3.00	12.29 ± 0.95^C^	11-13
***C. impunctatus***	6	11	2.64	0.00	0.00	0.00	0.00	0.00	0.00	0.00	0.67	0.44	1.11	1.78	2.78	9.33 ± 1.32^B^	7-12
***C. grisescens***	5	10	3.80	0.00	0.00	0.00	0.00	0.00	0.00	0.20	1.10	1.30	1.10	2.60	2.20	12.30 ± 1.83^C^	9-15
***C. kalix***	5	10	3.00	0.00	0.00	0.00	0.00	0.00	0.00	0.00	1.00	0.80	1.00	1.90	3.00	10.70 ± 0.82^B^	9-12

Total number of sensilla on the flagellum and minimum (MIN) and maximum (MAX) munmer of antennal senisilla. N_1_ = number of specimens; N_2_ = number counted. Means with the same letters are not significant different.

Two functional spermatheca, a third rudimentary, as well as a sclerotized ring are found in the abdomen. The functional spermatheca are ovoid and with a short neck. The sizes of the two functional spermatheca are almost equal and spermatheca ratio (S/R) is only different from *C. pulicaris*, *C. punctatus* and *C. kalix* (Table 5).

### Description of *Culicoides selandicus sp. nov*

Up till now only females of this species are known and therefore the following characteristics apply to females only. *Culicoides selandicus* have similarities to *C. punctatus*, and *C. newsteadi* and *C. kalix*.

The length of the wing is 1,339 ± 33 μm, which is the same size as *C. newsteadi* and *C. impunctatus* (Table 5). The shape of the dark hour-glass mark in the middle of cell r_5_ is broken and broadest above the longitudinal fold above M_1_ (Figure 3b). The dark areas on the wings are extensive and surrounding vein M_1_ and sometimes M_2_. Sometimes small pale spots are found at the tips of vein M_1_ and M_2_. Wings are with a large dark spot in cell cu separated from the dark areas bordering Cu_1_ and Cu_2_. The species can be confused with *C. newsteadi* due to the extensive dark areas on the wings, but can be distinguished from this by different forms of the maxillary palp segments (Figure 3, Table 4).

The eyes are contiguous and the length of contact is greater than one ocellus and only significantly different from *C. punctatus*, *C. newsteadi*, *C. impunctatus* and *C. grisescens* (P_K-WC_ < 0.05) (Table 6). The average length of antennal flagellum is 616.3 ± 9.9 μm, which is not significantly different from *C. newsteadi* (Table 3). The antennal ratio (AR) 1.12 ± 0.04 is significantly higher than in *C. newsteadi*, but of the same magnitude as in *C. punctatus, C. deltus, C. grisescens* and *C. kalix* (Table 3). The average number of sensillae is of the same magnitude as in *C. punctatus* and *C. grisescens,* but higher than the number found in *C. newsteadi* (Table 7). The third segment of the antenna has in average 4.1 sensillae, whereas *C. punctatus* and *C. kalix* have an average number of 3.4 and 3.0, respectively (Table 7). The length and shape of the third segment of the maxillary palp (PR = 3.2 ± 0.3) is more slender than that of *C. newsteadi* and the other species, but with the shape not significantly different from *C. grisescens* (Table 4). The second segment of the maxillary palp is as long as the third segment of the maxillary palp. The P 3/2 ratio of (length of third palp divided by the second) thus differs from all species except *C. punctatus* (Table 4). Segments four and five of the maxillary palp are of the same length. This is different from both *C. newsteadi* and *C. kalix*, where the fifth segment of the maxillary palp is longer than the fourth segment (Table 4). The numbers of mandibular and maxilla teeth are 15.0 ± 1.0 and 19.6 ± 1.5, respectively (Table 6). The ratio mandibular *vs*. maxillary teeth is 1.31 ± 0.12 is significantly higher (P_K-W_ <0.05) than in *C. kalix,* but of the same order of magnitude as in *C. punctatus* (1.26 ± 0.15) (Table 6).

The head/proboscis ratios (1.16 ± 0.06) show that *C. selandicus* is different from *C. newsteadi* (1.32 ± 0.13) and *C. kalix* (1.29 ± 0.07), but the ratios are of same magnitude as in *C. punctatus* (1.19 ± 0.07) (Table 5).

### Description of *Culicoides kalix sp. nov*

Up till now only females of this species are known and the following characteristics only apply to females. *C. kalix* has similarities to the following species *C. punctatus*, and *C. newsteadi* and *C. selandicus*.

Length of wing is 1,423 ± 39 μm. The shape of the dark hour-glass formed mark in the middle of r_5_ is broken and broadest above the longitudinal fold above M_1_ (Figure 3c). The dark areas on the wings are extensive and surrounding vein M_1_ and M_2_. Wings have a large dark spot in cell cu which is separated from the dark areas bordering Cu_1_ and Cu_2_. The species can be confused with *C. newsteadi* and *C. selandicus* due to the extensive dark areas on the wings. It can be distinguished from *C. newsteadi* by a significant more slender third palp segment (P_K-W_ <0.05) (Table 4). It can be distinguished from *C. selandicus* by a longer second palp segment than third palp segment (Table 4).

The eyes are continuous and the length of contact is greater than one ocellus and thus significantly different from *C. newsteadi* (Table 6). The average length of antennal flagellum is 646 ± 14 μm. The antennal ratio (AR) 1.13 ± 0.04 is significantly higher than in *C. newsteadi* (P_K-W_ < 0.05), but of the same magnitude as in *C. punctatus* and *C. selandicus* (Table 3). The average number of sensillae is 10.70 ± 0.82, which is lower and significantly different from *C. punctatus* and *C. selandicus* (P_K-W_ <0.05)*,* but a higher number than found in *C. newsteadi* (Table 7). The third segment of the antenna has an average of 3.0 sensillae comparable to *C. punctatus* with an average number of 3.4, but different from *C. selandicus* with an average of 4.1 and *C. newsteadi* with 2.9 sensillae (Table 7). The shape of the third segment of the maxillary palp (PR 2.9 ± 0.2) is different from *C. newsteadi* and *C. selandicus* (Table 4). The second maxillary palp segment is longer than the third (the P 3/2 ratio) and thus differs from both *C. selandicus* (second and third segment of equal length) and *C. newsteadi* (third segment longer than second segment) (Table 4). Segment four and five of the maxillary palp are of very different lengths, which separate the species from *C. selandicus*, where both segments are of equal length (Table 4). The numbers of mandibular and maxilla teeth are 12.8 ± 0.63 and 14.90 ± 1.5, respectively. This M/M ratio mandibular *vs.* maxillary to teeth (1.17 ± 0.12), is of the same order of magnitude as in *C. newsteadi* (1.18 ± 0.20), but lower than in *C. selandicus* (1.31 ± 0.12) and *C. punctatus* (1.26 ± 0.14) (Table 6). The head/proboscis ratios show that *C. kalix* (1.29 ± 0.07) is different from *C. selandicus* (1.16 ± 0.06) and *C. punctatus* (1.19 ± 0.07), but the ratios are of the same magnitude as in *C. newsteadi* (1.32 ± 0.13) (Table 5).

Two functional and a rudimentary third spermatheca as well as a sclerotized ring are found in the abdomen. The shape of the functional spermatheca is ovoid and provided with a short neck. The two spermatheca are different in size (Table 5).

## Conclusion

The females of three new species, *C. boyi, C. selandicus, C. kalix* are described. Interspecies differences across *Culicoides* subgenera using COI barcode approaches 20%, whereas intraspecies differences are below 4% and in most cases below 1%.

There has been a very wide collection of biting midges over a wide geographical area in many localities in Denmark and Sweden. Survey collections of biting midges at this level have never been done previously and had the trapping sites been fewer, it is doubtful whether the three new *Culicoides* species described here had been discovered. They are found at a single location or in close proximity. They are not found widely distributed, as most well-known biting midge species. This poses a question if the subgenus *Culicoides* is composed of a few species distributed over a large geographical area and many “local” species that occur on a few locations only.

## Additional files

Additional file 1: Table S1. Overview of the *Culicoides* COI barcode sequences of three Palaearctic subgenera; *Avaritia*, *Culicoides* and *Monoculicoides* compared. Additional file 2: Table S2. Interspecies differences of *Culicoides* COI sequences published in GenBank.
