# Supplementary material for: Delineation of Culicoides species by morphology and barcode exemplified by three new species of the subgenus Culicoides (Diptera: Ceratopogonidae) from Scandinavia
Source: Parasit Vectors. 2015 Mar 10;8:151. doi: 10.1186/s13071-015-0750-4 (PMC4372322; doi:10.1186/s13071-015-0750-4)
Supplement: Additional file 1: Table S1. — Overview of the Culicoides COI barcode sequences of three Palaearctic subgenera; Avaritia, Culicoides and Monoculicoides compared. [file 13071_2015_750_MOESM1_ESM.docx]

**Table S1**. Overview of the *Culicoides* COI barcode sequences of three Palaearctic subgenera; *Avaritia*, *Culicoides* and *Monoculicoides* compared. Accession numbers are grouped by countries: AL=Algeria, BG=Bulgaria, CH=Switzerland, DK=Denmark, GR=Greece, FR=France; IT=Italy, MO=Morocco, PO=Portugal, SW=Sweden, UK=United Kingdom.

| Species | GenBank |
| --- | --- |
| Subgenus: *Avaritia* |  |
| *C. chiopterus* | CH: HQ824396-412  DK: JQ683259-262  SW: JQ620044-048, JQ978440  UK: AM236747-751 |
| *C. dewulfi* | CH: HQ824413-416  DK: JQ683263  FR: HM022876-81  SW: JQ620063-67, JQ978428  UK or GR: AM236672-707 |
| *C. imicola* | AL: EU189055-057  GR: AJ549388-392  ES: AF080527-30, AF080536-540, AF083044, AJ867227-228, DQ871030  FR: HQ824456  IS: AF078097-100, AF080531-535, JN545055-56 AJ549393-414, AJ867223  IT: AJ867224-225, AJ867232-234  MO: AJ867226  PO: AF079975-979, AJ549415-426, AJ867229-231 |
| *C. obsoletus* | BG: AM23667-670  CH: HQ824371-380  DK: JQ683284-295  ES: DQ162808-816, JQ740594-596  FR: HM022792-HM022856  SW: JQ620130-140, JQ978426, JQ978438, JQ978448, JQ978451, JQ978462-463, JQ978465-466  UK: AM236652-666, AM236671 |
| *C. scoticus* | CH: HQ824385-395  DK: JQ683353-361  GR: AM236627, AM236651  FR: HM022857-875  ES: DQ162804-807  MO: AM236628-629  SW: JQ620202-210, JQ978417-418, JQ978425, JQ978427, JQ978429-432, JQ978458, JQ978460  UK: AM236625-626, AM2366230-250 |
| Subgenus: *Culicoides* |  |
| *C. deltus* | CH: HQ824454-455  DK: JF766300-309, JF766358 |
| *C. fagineus* F1 | ES: GQ338928-930 |
| *C. fagineus* F2 | ES: GQ338931-934 |
| *C. flavipulicaris* | ES: GQ338923-924 |
| *C. grisescens* | CH: HQ824446-447  DK: JF766302, JF766314-318  SW: JQ620086-091, JQ978433, JQ978439, JQ978464  UK: AM236726-732 |
| *grisescens* G2 | CH: HQ824448-453 |
| *C. impunctatus* | DK: JF766310-313, JQ683267  SW: JQ620092-096, JQ978444  UK: AM236717-725 |
| *C. lupicaris* | CH: HQ824424-433  ES: GQ338905-909 |
| *C. lupicaris* L2 | SW: JQ620103-109, JQ978452 |
| *C. newsteadi* | IT: AM236738-746 |
| *C. newsteadi* N1 | ES: GQ338915 |
| *C. newsteadi* N2 | ES: GQ338916-920 |
| *C. newsteadi N3*  (C. *halophilus*) | DK: JF766297-299, JF766319, JF766322-323, JF766327  ES: GQ338921-922  SW: JQ620112-119 |
| *C. newsteadi* N4 (Kalix *= C. kalix*) | SW: JF766328-332, JQ620120-124 |
| *C. newsteadi* N5  (dk3 = *C. selandicus*) | DK: JF766320, JF766324 JQ620125-126, JQ978445 |
| *C. pulicaris* | CH: HQ824417-423  DK: JF766333-345, JF766359-365  ES: GQ338912-914  SW: JF766350, JF766352-354, JQ620171-183, JQ978434, JQ978437  UK: AM236708-716 |
| *C. pulicaris* P3 | ES: GQ338910-911 |
| *C. pulicaris*  (dk1 = *C. boyi*) | DK: JF766293-296, JF766321, JF766346-349 |
| CH | CH: HQ824434-445, |
| *C. punctatus* | CH: HQ824499-507  DK: JF766325-326, JF766343, JF766355-357, JF766364, JQ683306-313, ES: GQ338893-904  SW: JF766351, JQ620184-188, JQ978419-424, JQ978435, JQ978441, JQ978449-450, JQ978453, JQ978455-457  UK: AM236733-737 |
| *C. subfagineus* | ES: GQ338925-927 |
| Subgenus: *Monoculicoides* |  |
| *C. nubeculosus* | DK: JQ683275-283  SW: JQ620127-129 |
| *C. puncticollis* | DK: JQ683314-333 |
| *C. riethi* | DK: JQ683335-348  SW: JQ620195-196 |
| *C. stigma* | SW: JQ620229-234 |
