# Supplementary material for: Delineation of Culicoides species by morphology and barcode exemplified by three new species of the subgenus Culicoides (Diptera: Ceratopogonidae) from Scandinavia
Source: Parasit Vectors. 2015 Mar 10;8:151. doi: 10.1186/s13071-015-0750-4 (PMC4372322; doi:10.1186/s13071-015-0750-4)
Supplement: Additional file 2: Table S2. — Interspecies differences of Culicoides COI sequences published in GenBank. [file 13071_2015_750_MOESM2_ESM.docx]

**Table S2.** Interspecies differences of *Culicoides* COI sequences published in GenBank. Accesion numbers from GenBank is presented after species names in the table.

| Species | GenBank Acc. | Divergence from type-species C. punctatus: GenBank AM236733 |
| --- | --- | --- |
| Subgenus: *Avaritia* |  | % |
| *C. chiopterus* | AM236747 | 18 |
| *C. dewulfi* | AM236672 | 21 |
| *C. imicola* | AF069231 | 18 |
| *C. obsoletus* | AM236652 | 17 |
| *C. scoticus* | AM236625 | 17 |
|  |  |  |
| Subgenus: *Beltranmyia* |  |  |
| *C. circumscriptus* | HQ824460 | 21 |
| *C. manchuriensis* | JQ620110 | 21 |
| *C. salinarius* | JQ620197 | 21 |
| *C. sphagnumensis* | JQ620224 | 21 |
|  |  |  |
| Subgenus: *Culicoides* |  |  |
| *C. deltus* | JF766300 | 17 |
| *C. fagineus* | GQ338928 | 19 |
| *C. flavipulicaris* | GQ338923 | 17 |
| *C. grisescens* | AM236726 | 17 |
| *C. impunctatus* | AM236717 | 18 |
| *C. lupicaris* | HQ824424 | 16 |
| *C. newsteadi* | AM236738 | 16 |
| *C. pulicaris* | AM236708 | 17 |
| *C. punctatus* | AM236733 | 0 |
| *C. subfagineus* | GQ338925 | 17 |
|  |  |  |
| Subgenus: *Monoculicoides* |  |  |
| *C. nubeculosus* | JQ683275 | 19 |
| *C. puncticollis* | JQ683314 | 19 |
| *C. riethi* | JQ683335 | 19 |
| *C. stigma* | JQ620229 | 17 |
|  |  |  |
| Subgenus: *Oecacta* |  |  |
| *C. alazanicus* | JQ620023 | 19 |
| *C. albicans* | JQ620030 | 19 |
| *C. brunnicans* | JQ6200033 | 17 |
| *C. clastrieri* | JQ6200055 | 21 |
| *C. clintoni* | JQ6200062 | 18 |
| *C. comosioculatus* | HQ824466 | 20 |
| *C. duddingstoni* | JQ6200072 | 16 |
| *C. festivipennis* | JQ6200081 | 21 |
| *C. furcillatus* | JQ683266 | 17 |
| *C. jurensis* | HQ824486 | 20 |
| *C. kibunensis* | JQ683273 | 20 |
| *C. pictipennis* | JQ620161 | 16 |
| *C. poperinghensis* | JQ683298 | 20 |
| *C. simulator* | JQ620219 | 16 |
| *C. truncorum* | JQ620239 | 21 |
| *C. vexans* | JQ683362 | 17 |
|  |  |  |
| Sugenus: *Silvaticulicoides* |  |  |
| *C. achrayi* | JQ620014 | 18 |
| *C. fascipennis* | JQ620074 | 17 |
| *C. pallidicornis* | JQ620147 | 17 |
| *C. subfasciipennis* | JQ620235 | 20 |
|  |  |  |
| Subgenus: *Wirthomyia* |  |  |
| *C. reconditus* | JQ620189 | 17 |
| *C. segnis* | JQ620211 | 19 |
